# Effects of training Community Health Agents on dementia: a quasi-experimental study

**DOI:** 10.1590/0034-7167-2023-0027

**Published:** 2024-03-15

**Authors:** Aline Cristina Ramos Coelho, Edmir Batista da Silva Cruz, Helena Zacharias Radicchi, Emanuela Bezerra Torres Mattos, Marcia Maria Pires Camargo Novelli, Felipe Granado de Souza, Rubens Goulart, Corina Lopes Ribeiro

**Affiliations:** I Universidade Federal de São Paulo. Santos, São Paulo, Brazil; II Secretaria Municipal de Saúde de Santos. Santos, São Paulo, Brazil.; III Ambulatório de Especialidades. Santos, São Paulo, Brazil.

**Keywords:** Training, Community Health Workers, Dementia, eHealth Strategies, Aging, Capacitación, Agentes Comunitarios de Salud, Demencia, Estrategias de eSalud, Envejecimiento, Capacitação, Agentes Comunitários de Saúde, Demência, Estratégias de eSaúde, Envelhecimento

## Abstract

**Objectives:**

to evaluate the knowledge of Community Health Agents about dementia before and after the training workshop for detecting signs of the disease.

**Methods:**

a quasi-experimental study with 33 community agents, in which sociodemographic information and knowledge about dementia were collected and assessed using the Alzheimer’s Disease Knowledge Scale before and after the workshop. There were 10 weekly, online, synchronous meetings. The Student’s t-test for related samples was used, and the effect size was calculated.

**Results:**

while the average score on the initial assessment, using the measurement instrument, was 16.3, it was 21.24 in the final assessment. An increase in the scale score was observed after participating in the workshop, with a value of 4.94.

**Conclusions:**

it is urgent to invest in the ongoing education of these professionals for greater awareness in the timely detection of dementia cases in primary care and awareness of potentially modifiable factors.

## RESUMO

**Objetivos:**

avaliar o conhecimento dos Agentes Comunitários de Saúde sobre demência antes e após a oficina de capacitação para detecção de sinais da doença.

**Métodos:**

estudo quase-experimental com 33 agentes comunitários, no qual foram coletadas informações sociodemográficas e de conhecimento sobre demência, avaliadas pela *Alzheimer’s Disease Knowledge Scale*, no pré e pós-oficina. Houve 10 encontros semanais, online e síncronos. Utilizou-se o teste t de *Student* para amostras relacionadas e calculou-se o tamanho do efeito.

**Resultados:**

enquanto a média de pontos na avaliação inicial, por meio do instrumento de medida, foi de 16,3, na avaliação final foi de 21,24. Observou-se aumento na pontuação na escala após a participação na oficina, com um valor de 4,94.

**Conclusões:**

é urgente investir na educação permanente desses profissionais para maior conscientização na detecção oportuna de casos de demência ainda na atenção básica e conscientização de fatores potencialmente modificáveis.

## RESUMEN

**Objetivos:**

evaluar el conocimiento de los Agentes Comunitarios de Salud sobre la demencia antes y después de un taller de capacitación para la detección de signos de la enfermedad.

**Métodos:**

estudio cuasi experimental con 33 agentes comunitarios, en el que se recopilaron datos sociodemográficos y conocimiento sobre la demencia, evaluados mediante la Escala de Conocimiento de la Enfermedad de Alzheimer, antes y después del taller. Hubo 10 sesiones semanales en línea y sincrónicas. Se utilizó la prueba t de *Student* para muestras relacionadas y se calculó el tamaño del efecto.

**Resultados:**

mientras que la puntuación promedio en la evaluación inicial a través del instrumento de medición fue de 16,3, en la evaluación final fue de 21,24. Se observó un aumento en la puntuación de instrumento después de participar en el taller, con un valor de 4,94.

**Conclusiones:**

es urgente invertir en la educación continua de estos profesionales para aumentar la conciencia en la detección oportuna de casos de demencia en atención primaria y la concienciación sobre factores potencialmente modificables.

## INTRODUCTION

In the United States and worldwide, due to the aging population, there is a growing increase in cases of dementia^(1)^. Currently defined by the Diagnostic and Statistical Manual of Mental Disorders (DSM-5) as Major Neurocognitive Disorder (MND), it is a clinical syndrome characterized by cognitive and functional impairment, leading to gradual dependence on the care of others as the disease progresses^(2)^.

Estimates from Alzheimer’s Disease International (ADI)^(3)^ indicate that more than 50 million people worldwide have some form of dementia, with 60% residing in lowand middle-income countries. It is projected that this number will rise to 82 million by 2030 and 152 million by 2050, with a current annual cost of $1 trillion, potentially doubling by 2030.

Prevalence and incidence projections suggest that the number of people with MND will continue to grow, especially among the older elderly, potentially tripling by 2050, primarily in lowand middle-income countries^(4)^. People with dementia, due to their clinical condition, frequently use primary healthcare services^(5)^. However, research points to significant barriers preventing healthcare teams from providing timely diagnosis and treatment for dementia at this level of care.

Research conducted in Australia identified key barriers, including a lack of knowledge and skills, as well as healthcare professionals’ uncertainty in identifying symptoms and making referrals^(5)^. These issues delay diagnosis and hinder the timely implementation of pharmacological and non-pharmacological treatments, contributing to late diagnosis, high hospitalization rates, and early use of long-term care facilities^(5-6)^.

To promote better care, studies emphasize the need to invest in the training of professionals from various healthcare fields (physicians, nurses, occupational therapists, nutritionists, physiotherapists, psychologists, social workers, and community health workers) to ensure collaborative interprofessional practices centered on elderly individuals with dementia and their caregivers or family members^(7)^.

In Germany, Dem-NetD, an interdisciplinary center that provides a network of dementia services, analyzed 13 different care networks to identify motivating or inhibiting factors for successful primary care. Among the motivating factors, they found the overcoming of competitive relationships between various professional areas involved in treatment and care, the possibility of integrating services with community care, access to medical services and rehabilitation professionals, the quality of hospitalization and nursing care, self-help organizations, pharmaceutical monitoring, and local public policies aimed at maintaining or improving the quality of life and reducing caregiver burden. Among the inhibiting factors, they mentioned the diverse and complex network of dementia care, which varies greatly depending on the professional area of the coordinator of each network, as well as the specific objectives of each coordinator, funding sources, and cooperation structures involved. Regardless of the identified factors, the study suggests that cooperative models involving various fields of knowledge were able to promote the integration of dementia into society by providing information to overcome stigma and reduce social barriers between healthcare professionals, patients, and the community^(7-8)^.

The World Health Organization (WHO) has launched a global challenge for the development and implementation of dementia action plans. More recently, the Pan American Health Organization (PAHO) issued a document signed by all countries in Latin America, stating that all countries are compelled to build dementia-focused programs as public measures to address the increasing prevalence rates of dementia in the region^(9)^.

Meanwhile, in Europe, countries have already implemented their national plans and have converged toward a global commitment to ensure a proactive approach to the diagnosis and treatment of dementia. This involves expanding the public healthcare network and developing health policies, as well as programs and strategies to respond to the broad and complex impact of this disease on societies^(9-11)^. To achieve these goals, the WHO has suggested seven priority action domains: awareness of the disease, early diagnosis, high-quality continuous care services, support, workforce training, prevention, and research tailored to local and cultural needs. These domains should be addressed and discussed by local governments^(1,3)^.

The important warning that only one-fifth of diagnoses in developed countries are routinely recognized and documented in primary care, along with the limited availability of adequately qualified healthcare professionals to deal with the complex demands of individuals with MND and their families, reinforces the need for investment in the training of primary healthcare professionals to contribute to the development of a national action plan^(1)^.

In Brazil, the main healthcare policy is the Unified Health System (SUS in portuguese), in which primary care serves as the entry point for individuals into the system. In 2005, the Ministry of Health defined the Health Commitment Agenda, which encompasses three pillars: the Pact for the SUS, the Pact for Life, and the Management Pact. The Pact for Life places special emphasis on improving access and the quality of services provided in the healthcare system, with a focus on strengthening and strategically qualifying the Family Health program to offer special care strategies related to the aging process, among other strategies^(12)^.

The National Health Promotion Policy (PNPS) advocates for the qualification of professionals to work in the field of health promotion, information, communication, and popular education within the Family Health Strategy and the Community Health Agents Program. This policy values the inclusion of training in subjects related to health promotion within the SUS^(12)^. Additionally, Decree No. 3,189 of October 4, 1999, provides guidelines for the work of CHAs in carrying out disease prevention and health promotion activities through individual and collective educational actions in households and communities under competent supervision^(13)^.

## OBJECTIVES

To evaluate the knowledge of Community Health Agents regarding dementia before and after participating in a training workshop for detecting signs of the disease.

## METHODS

### Ethical considerations

This pilot study was submitted to and approved by the Municipal Health Department of Santos and the Ethics Committee for Human Research at the Federal University of São Paulo/Campus Baixada Santista. The study was conducted in accordance with the principles outlined in the Declaration of Helsinki by the World Medical Association (1964, revised in 1975, 1983, 1989, 1996, and 2000) and Resolution 510/2016 of the National Health Council.

### Study design, period and location

This study follows a quasi-experimental design (pre-test and post-test) aimed at assessing the knowledge of CHAs regarding dementia before and after their participation in a training workshop. In this study, only one group was used, which consisted of the CHAs who attended the workshop. In a quasi-experimental design, there is no control group for direct comparison. Instead, the study compares the knowledge of CHAs before and after the intervention, using the same group as its own reference. This approach allows us to evaluate whether the training workshop significantly improved the CHAs’ knowledge about dementia.

The registration form was initially shared with the coordinators of the Basic Health Units in the city of Santos in January/February 2021. This online form included the informed consent form (ICF) for participants, in which the research objectives and ethical principles were explained. Additionally, sociodemographic information, questions related to professional roles, and information about any previous training related to aging and/or dementia were collected. The research was conducted from April 2020 to May 2021.

### Sample; inclusion and exclusion criteria

The participants in this study were CHAs currently employed by the Health Department of the city of Santos, São Paulo. The selection of CHAs was carried out by the city’s Health Department, including all individuals recommended by the department. Inclusion criteria required participants to be CHAs in the city of Santos, available for the 10 sessions, have internet access, and possess electronic equipment. The exclusion criterion was having more than two absences, which equated to more than 25%.

### Study Protocol

The process, including both the pre-workshop and post-workshop phases, consisted of 10 weekly meetings, each lasting 1.5 hours, held in the evening via the Google Meet platform. In the first meeting, the Alzheimer’s Disease Knowledge Scale (ADKS) (Pre-Workshop) in digital format was provided to assess the level of knowledge about dementia before the workshop. This scale has been translated into Portuguese^(14)^. It is a dichotomous scale (True or False) composed of 30 questions divided into seven subscales: Impact on Life (questions 1, 11, 28); Risk Factors (2, 13, 18, 25, 26, and 27); Disease Course (3, 8, 14, 17); Assessment and Diagnosis (4, 10, 20, 21); Care (5, 6, 7, 15, 16); Treatment and Symptom Management (9, 12, 24, and 29); and Symptoms (19, 22, 23, 30)^(15)^.

Following the authors’ recommendations, the “I don’t know” option was included, and the response options True or False were used instead of Yes or No. This scale does not have a specific cutoff point, and for evaluation purposes, one point was assigned for each correct response. Thus, the score ranges from 0 to 30 points, with a higher score indicating a better understanding of dementia, especially Alzheimer’s disease. Based on the seven premises of the World Health Organization^(1)^ and the content proposed in the Interprofessional Education module described by Dreier-Wolfgramm et al.^(7)^, central topics were addressed in meetings from the 2nd to the 9th, including: key concepts in dementia; pharmacological and non-pharmacological treatment; prevention and risk factors; disease stages and common subtypes; cognitive and behavioral changes; cognitive and functional screening instruments; referral and counter-referral network in the Unified Health System of Santos; and, finally, stigma.

After each of these 8 thematic meetings, an online quiz with multiple-choice questions related to the topic discussed was administered to complement the knowledge assessment. All participants had access to reading materials and didactic support materials related to the weekly topics. In the final meeting, the ADKS was re-administered (Post-Workshop), followed by a discussion of the questions evaluated by the scale to clarify any possible doubts.

### Results Analysis

All online assessment instruments were recorded in an Excel spreadsheet for statistical analysis. In the statistical analysis, data were described with categorical variables presented in absolute and relative frequencies, and numerical variables presented as mean and standard deviation. To evaluate the effect of the training workshop, the Shapiro-Wilk test was initially used to check if the data distribution followed a normal distribution, which was found to be satisfactory. Subsequently, the paired Student’s t-test was used, and effect size was calculated. The Delta ADKS was also calculated, representing the difference between the post-intervention and pre-intervention assessments, with the aim of studying the relationship between the quiz and the change in ADKS score, measured by Pearson’s linear correlation coefficient. A significance level of 0.05 was considered. The software used was R Core Team^(16)^. An adaptation of the CONSORT 2010 checklist guidelines was made since there was no randomization^(17)^.

## RESULTS

A total of 43 CHAs registered, of whom 33 were affiliated with 10 Basic Health Units (BHUs) out of the 32 in the city of Santos, and they completed the required minimum workload (17 hours out of a total of 20). Among the dropouts, 3 did not attend any of the meetings, and 7 cited difficulties with the workshop schedule and COVID-19 contamination as reasons.

Among the participants, females predominated (78.8%), with a marital status of married (39.4%), and a completed high school education (45.5%). 66.7% have been in their role for more than 16 years and received training for their position (97%). 76% reported having received training/capacity building in the field of aging, while 79% had not received any training related to dementia.

Among the CHAs, 94% provide care in their territory to families with individuals over 65 years of age, dealing with issues such as hypertension (100%), diabetes (97%), sequelae from strokes (35%), and dementia (27%).

Based on the responses obtained in the pre-workshop ADKS application, only 15.2% of the CHAs answered the question “Having high cholesterol can increase the risk of a person developing Alzheimer’s disease” correctly, and 9.1% answered the question “Having high blood pressure can increase the risk of a person developing Alzheimer’s disease” correctly, both related to the “risk factors” subscale. This low percentage of correct answers reflects limited knowledge about the risk factors associated with dementia. In addition to the risk factor, the subscale assessing knowledge about “care” in dementia also showed a low score.

In the pre-workshop phase, all participants completed the Online Sociodemographic Questionnaire, which also included questions related to the developed theme, in addition to the Alzheimer’s Disease Knowledge Scale (ADKS) in digital format to assess the participants’ prior knowledge about dementia and Alzheimer’s disease. The overall mean score on the pre-workshop ADKS was 16.30, with a standard deviation of 3.46. This result can be considered low because, considering the standard deviation, this score is less than half of the total knowledge level, indicating a low level of knowledge among the participants (Table 1).

**Table 1:** Alzheimer’s Disease Knowledge Scale Results by Subscale in the Pre-Workshop Assessment

Subscale	Questions	Correct Answers
Impact on Life	1. People with Alzheimer’s disease are particularly prone to depression.	66.7%
	2. It is scientifically proven that mental exercise can prevent a person from getting Alzheimer’s disease.	63.6%
	3. After the onset of Alzheimer’s disease symptoms, the average life expectancy is 6 to 12 years.	78.8%
Risk Factors	4. When a person with Alzheimer’s disease becomes agitated, a medical examination can reveal other health problems that have caused the agitation.	9.1%
	5. People with Alzheimer’s disease do better with simple instructions, taking one step at a time.	6.1%
	6. When people with Alzheimer’s disease begin to have difficulty taking care of themselves, caregivers should take over immediately.	15.2%
	7. If a person with Alzheimer’s disease becomes alert and agitated at night, a good strategy is to try to ensure that the person engages in a lot of physical activity during the day.	48.5%
	8. In rare cases, people have recovered from Alzheimer’s disease.	9.1%
	9. People whose Alzheimer’s disease is not yet severe may benefit from psychotherapy for depression and anxiety.	45.5%
Course of the Disease	10. If memory problems and confused thinking arise suddenly, it is likely due to Alzheimer’s disease.	21.2%
	11. Most people with Alzheimer’s disease live in nursing homes.	75.8%
	12. Malnutrition can worsen the symptoms of Alzheimer’s disease.	60.6%
	13. People in their 30s may have Alzheimer’s disease.	84.8%
Assessment and Diagnosis	14. A person with Alzheimer’s disease is increasingly likely to fall as the disease worsens.	60.6%
	15. When people with Alzheimer’s disease repeat the same question or story several times, it is helpful to remind them that they are repeating themselves.	60.6%
	16. Once people have Alzheimer’s disease, they are no longer able to make conscious decisions about their own care.	42.4%
	17. Eventually, a person with Alzheimer’s disease will need 24-hour supervision.	81.8%
Care	18. Having high cholesterol can increase a person’s risk of developing Alzheimer’s disease.	75.8%
	19. Tremor or shaking of the hands or arms is a common symptom in people with Alzheimer’s disease.	12.1%
	20. Symptoms of severe depression can be confused with symptoms of Alzheimer’s disease.	45.5%
	21. Alzheimer’s disease is a type of dementia.	39.4%
	22. Trouble handling money or paying bills is a common early symptom of Alzheimer’s disease.	30.3%
Treatment and Symptom Management	23. A symptom that can occur with Alzheimer’s disease is believing that other people are stealing their things.	69.7%
	24. When a person has Alzheimer’s disease, using reminders is a crutch that can contribute to decline.	63.6%
	25. Prescription medications that prevent Alzheimer’s disease are available.	45.5%
	26. Having high blood pressure can increase a person’s risk of developing Alzheimer’s disease.	93.9%
Symptoms	27. Genes may only be partially responsible for the development of Alzheimer’s disease.	81.8%
	28. It is safe for people with Alzheimer’s disease to drive as long as they have a companion in the car all the time.	57.6%
	29. Alzheimer’s disease has no cure.	84.8%
	30. Most people with Alzheimer’s disease remember recent events better than things that happened in the past.	100,0%

Furthermore, an Online Questionnaire was administered to evaluate the CHAs’ perception of their own knowledge about dementia. 30.5% of the CHAs reported having good general knowledge, 60% reported having reasonable knowledge, and 9% reported having poor knowledge.

To assess knowledge about referral services in the primary care network of the municipality, 42.5% of the participants claimed to know to which service a user with any sign of possible dementia should be referred after diagnosis. Only 8.3% correctly identified the Specialties Outpatient Clinic, while the others believed that the appropriate referral should be to the Psychosocial Care Center (CAPS) or the Alcohol and Drugs Psychosocial Care Center (CAPS-AD). These results reflect the lack of knowledge among primary care professionals about referral services in the healthcare network^(5)^, as well as the stigma associated with dementia when professionals and members of the community in general associate dementia symptoms with those common in patients with psychiatric illnesses such as schizophrenia^(18-20)^.

To compare the ADKS scores before and after the training workshop, the paired Student’s t-test was used. The results of the means, differences, and the test outcome are presented in Table 2. There was an average increase in ADKS scores after the CHAs participated in the workshop, with a value of 4.94, 95% CI (3.58; 6.30). This difference was statistically significant, with a p-value of < 0.001 (Table 2).

**Table 2:** Descriptive Measures and Comparison of Alzheimer’s Disease Knowledge Scale Scores Pre and Post-Workshop

ADKS 1 Mean (SD)	ADKS 2 Mean (SD)	Difference (95% CI)	*p* value	ES (95% CI)
16.3 (3.46)	21.24 (3.29)	4.94 (3.58;6.30)-6	<0.001	1.29 (0.83;1.77)

*ADKS - Alzheimer’s Disease Knowledge Scale; SD - Standard Deviation; ES - Effect Size; CI - Confidence Interval.*

When assessing the correlation between Delta ADKS and each of the thematic quizzes outlined in Table 3, a moderate correlation was identified with quiz 3, focusing on dementia prevention (healthy habits: diet, physical activity, social engagement, and new learning) (r = 0.47). This correlation could be linked to the fact that this topic is shared among generalist healthcare practitioners, while the others are more closely connected to specific and distinctive facets of dementia.

**Table 3:** Pearson’s linear correlation coefficient, confidence interval, and p-value for the associations between the Delta Alzheimer’s Disease Knowledge Scale variable and the quiz variables

Variables		Coefficient	95% CI	*p* value
Delta ADKS	Dementia (definition, causes, prevalence, and incidence, risk factors)	0.34	(-0.03; 0.63)	0.069
Delta ADKS	Dementia (clinical assessment, diagnosis, prognosis, pharmacological and non-pharmacological treatment)	-0.12	(-0.46; 0.25)	0.524
Delta ADKS	Dementia prevention (healthy habits: diet, physical activity, social participation, and new learning)	0.47	(0.12; 0.72)	0.011
Delta ADKS	Stages of dementia and the most common subtypes	-0.21	(-0.56; 0.20)	0.308
Delta ADKS	Cognitive and behavioral changes impact on functionality	0.31	(-0.05; 0.60)	0.092
Delta ADKS	Application of cognitive screening instruments: Mini-Mental State Examination and Pfeffer Scale	0.05	(-0.31; 0.40)	0.783
Delta ADKS	Referral and counter-referral network (referral process)	0.27	(-0.10; 0.58)	0.144
Delta ADKS	Stigma	0.31	(-0.08; 0.62)	0.118

*IC - Intervalo de Confiança; ADKS - Alzheimer’s Disease Knowledge Scale.*

## DISCUSSION

The ADKS helps identify educational needs related to Alzheimer’s disease and is also recommended for assessing the effectiveness of training programs for primary healthcare professionals in this field^(14)^. Primary care plays a crucial role in managing chronic diseases like dementia because it can provide equitable access to community-based care and early interventions in the disease process, ensuring a better quality of life and health for the user and their caregiver/family.

However, research indicates that the high prevalence of late diagnoses and barriers to timely diagnosis result from a lack of preparedness for dementia care in professional education, a lack of knowledge and confidence in professional skills, diagnostic uncertainty, and the complexity of dementia, which often co-occurs with other comorbidities. Additionally, limitations in primary care infrastructure, such as limited time availability and access to healthcare professionals, contribute to these challenges^(9,20)^. Our results align with the “It’s Never Too Early. It’s Never Too Late” campaign recently launched by Alzheimer’s Disease International in September, globally recognized as World Alzheimer’s Month, dedicated to raising awareness about risk factors and 12 potentially modifiable factors, including diabetes, high blood pressure, physical inactivity, excessive alcohol consumption, obesity, smoking, social isolation, air pollution, head injury, depression, hearing difficulty, and low education levels^(21)^. This underscores the need for investment in training and qualifying healthcare professionals, as well as increasing public awareness through media campaigns targeting the general population.

In Brazil, Bill 4,364 of November 16, 2020, established the National Policy to Confront Alzheimer’s Disease and Other Dementias^(22)^. This action represents a significant step forward and reinforces the need for investment in healthcare professional training for early symptom diagnosis and the organization of healthcare services for patient follow-up and coordination across the healthcare network. This is an issue that affects not only medical professionals but all those working at this level of care. Although this pilot study targeted CHAs, international studies indicate that a lack of knowledge (training and specific dementia education) is common among all primary healthcare professionals. To implement interprofessional collaborative practice models, researchers suggest the prior use of assessment tools that identify personal and professional skills, as well as scales for measuring the level of knowledge of professionals involved in dementia care. This helps in creating personalized training models^(7,20,23-24)^.

In Macau, China, a study applied a Knowledge, Attitude, and Practice Questionnaire on dementia prevention care among primary care professionals. Out of 234 participants, including doctors and nurses working at this level of care, 34.2% believed that dementia is a normal part of the aging process, and 23.3% believed that hypertension and diabetes mellitus are not risk factors for dementia^(25)^. Lack of knowledge and the stigma associated with dementia appear to be common across different levels of care and various professional fields.

The absence of training and education focused on dementia can be one of the main factors related to the lack of awareness regarding the importance of prevention in reducing the incidence rates of new cases and delaying the onset of symptoms. Annual extensive campaigns on social media, in collaboration with federal, state, and municipal agencies, can enhance dementia prevention efforts. Health promotion activities in primary care position Community Health Workers (CHWs) in a privileged position with access to local communities. When trained, they can serve as information multipliers, raising awareness among populations about the need to reduce cardiovascular risk factors (hypertension, obesity, dyslipidemia, and diabetes), address psychological factors (depression, anxiety disorders, personality disorders, and sleep disturbances), adopt a healthy lifestyle that reduces alcohol and tobacco consumption, combat sedentary behavior, follow a low-carbohydrate diet, and promote rich social and cognitive stimuli^(1,26-27)^.

A study conducted in Australia assessed the effectiveness of hybrid training (online and in-person) on dementia for nurses working in primary care. The results identified higher scores for those who participated in the in-person format compared to those in the virtual format^(5)^. Nevertheless, both formats demonstrated effectiveness while highlighting advantages and disadvantages for each approach. While in-person training was considered long-term and costly due to rigorous logistics, online training offers greater flexibility at a lower cost. Regardless of the format, there is a reinforced need for ongoing follow-up and updates of these training programs to provide continuous, interdisciplinary, and personalized support tailored to the demands of each professional area and their role in the healthcare network^(5)^.

To implement the national dementia action plan in Brazil, CHWs should be considered a fundamental part of primary healthcare delivery since they are the primary link between the user and the healthcare network^(20)^. There are substantial benefits to early diagnosis and management of dementia^(18,27)^, as nearly two-thirds of people with dementia live and die with the condition without ever being diagnosed^(28)^. Estimates suggest that there are 1.6 million elderly individuals with dementia, and of these, 1.2 million remain undiagnosed^(9,28)^.

These data present significant challenges in actions aimed at increasing awareness and providing training for healthcare teams to diagnose and treat people with dementia^(9)^. Lack of knowledge about dementia among healthcare professionals can be a barrier to early assessment, timely diagnosis, referral, and access to support and services^(18)^, and continuing education can bring about considerable changes in dementia diagnosis and treatment^(5,29-30)^.

In May 2021, Alzheimer’s Disease International presented the 4th Impact Plan, mapping which stage (1 - no current contact with the government, 2 - no strategy or action plan, 3 - action plan is under development, 4 - action plan adopted with little or no funding, and 5 - action plan implemented) each of the ADI member countries is at. Globally, 40 countries have national dementia plans. However, 28 new plans are needed annually for the World Health Organization (WHO) to achieve the goal of 146 plans by 2025. 141 WHO member countries still do not have a plan, including Brazil^(3,11)^.

Based on shared publications recounting successful experiences from countries in implementing their action plans/strategies, funding these actions was crucial. In France, public and private funding supported the opening of caregiver training schools as well as healthcare professional training^(9)^.

As the largest country in Latin America with significant economic and social inequalities among regions, Brazil needs to invest in research to identify the specific needs of people with dementia, their caregivers or family members, and healthcare professionals. These insights can provide the groundwork for the implementation of the national action plan^(9)^.

### Study limitations

This study has some limitations that should be considered when interpreting its results. Firstly, the sample used was relatively small, which may limit the generalizability of the findings to a broader population. Additionally, the quasi-experimental design adopted, while valuable for identifying patterns and trends, does not allow for establishing definitive causality. Therefore, the relationship between the intervention and the improvement in dementia knowledge, although suggestive, requires more robust investigations for confirmation. Furthermore, the quasi-experimental approach may not capture all factors that can influence the outcomes.

### Contributions to the Public Health Field

By confirming the effectiveness of the training model focused on Community Health Workers, our data highlight the importance of continuous education for this group. It is suggested that investing in specific training programs for CHWs may result in a deeper awareness of dementia topics, contributing to the early detection of these conditions in primary care.

## CONCLUSIONS

An increase in ADKS scores was observed after participation in the workshop. Based on the results observed so far, there is a need for continued education investment for community health workers in the municipality to raise awareness of the topic and increase the detection of dementia cases in primary care. The results of this pilot study can be adapted for the training of professionals from other areas and regions in our country, and it may be interesting to analyze with a larger sample of participants.
